# Office-Based Case Finding for Chronic Obstructive Pulmonary Disease in Older Adults in Primary Care

**DOI:** 10.1155/2016/1083270

**Published:** 2016-06-22

**Authors:** Linda Lee, Tejal Patel, Loretta M. Hillier, James Milligan

**Affiliations:** ^1^Centre for Family Medicine Family Health Team, 10B Victoria Street South, Kitchener, ON, Canada N2G 1C5; ^2^Department of Family Medicine, Faculty of Health Sciences, McMaster University, Hamilton, ON, Canada; ^3^Schlegel-University of Waterloo Research Institute for Aging, Waterloo, ON, Canada; ^4^University of Waterloo School of Pharmacy, Waterloo, ON, Canada; ^5^St. Joseph's Health Care, London, ON, Canada; ^6^Aging, Rehabilitation and Geriatric Care Research Centre of the Lawson Health Research Institute, London, ON, Canada

## Abstract

*Background*. Chronic Obstructive Pulmonary Disease (COPD) is underdiagnosed in primary care.* Aim*. To explore the utility of proactive identification of COPD in patients 75 years of age and older in a Canadian primary care setting.* Methods.* Canadian Thoracic Society (CTS) screening questions were administered to patients with a smoking history of 20 pack-years or more; those with a positive screen were referred for postbronchodilator spirometry.* Results*. A total of 107 patients (21%), of 499 screened, had a 20-pack-year smoking history; 105 patients completed the CTS screening. Forty-four (42%) patients were positive on one or more questions on the screening; significantly more patients with a previous diagnosis of COPD (64%) were positive on the CTS compared to those without a previous diagnosis of COPD (30%). Of those who were not previously diagnosed with COPD (*N* = 11), four (36%) were newly diagnosed with COPD.* Conclusion*. A systematic two-stage method of screening for COPD, using CTS screening questions followed by spirometric confirmation, is feasible in the context of a busy primary care setting. More research is needed to assess the value of restricting screening to patients with a smoking history of 20 pack-years and on the sensitivity and specificity of these measures.

## 1. Introduction

Chronic Obstructive Pulmonary Disease (COPD) is associated with increasing economic and social burden and is a leading cause of morbidity and mortality worldwide [1]. In Canada, it is the fourth leading cause of death [2]. Worldwide, the prevalence of COPD is estimated at 9-10% based on physiologic studies and 3–8% based on physician or patient reported diagnosis or symptoms [3]. In Canada, prevalence is estimated to be within 3–12% [3]. COPD is a common condition among older adults. In one Ontario study, 58% of the individuals with COPD were between the ages of 35 and 64 years; however, 42% of the individuals were aged 65 years and older [4]. Similarly, a national surveillance study found that prevalence rates increased with age, increasing from 3.3% in those 50–59 years of age to 6.6% and 11.3%, respectively, in those 60–69 and 70–79 years of age [3]. This trend is evident globally, with prevalence estimates of COPD increasing with age from 2.7% in those under 40 years of age to 7.6% among 40–64 year olds and 15% among those aged 65 years and older [5]. Although the incidence of COPD is not well elucidated, it is important to note that, in a screening strategy aimed specifically at community-dwelling frail individuals 65 years and older, previously unrecognized COPD was detected in 16.8% of individuals [6].

A considerable body of literature illustrates the underdiagnosis of COPD with less than a quarter to half of cases of COPD diagnosed in primary care settings [7–11] and in population-based studies [12–14]. A retrospective audit of 38,859 charts of patients with COPD found that opportunities for diagnosis were missed in 85% of patients [15]. Recent studies have demonstrated feasibility and effectiveness of case finding for COPD in younger adults, with ages ranging up to 65 years in primary care using respiratory health-screening questionnaires and diagnostic spirometry in high-risk subjects [16–19]. Among frail older adults screening for dyspnea or reduced exercise tolerance was found, in one study, to lead to new diagnoses of COPD and heart failure; the average age of those who had dyspnea and/or reduced exercise tolerance was 75.5 years (±6.1) [6]. Overall, little is known about case finding for COPD among the elderly; it is likely that unrecognized COPD results in destabilization and crisis events leading to Emergency Department visits and hospitalization.

Persons with COPD account for disproportionately high health utilization; in Ontario one study [4] found that emergency department visits and hospitalization by persons with COPD were, respectively, 85% and 65% higher than the rest of the population and that those older than 65 years of age used more health services than those aged 35 to 64 years of age. When the COPD sample was stratified by incident (newly diagnosed) and prevalent (existing disease) cases, hospitalizations among the incident COPD population were 530/1000 person years, higher than the prevalent COPD population (495/1000 person years) and more than double that of the non-COPD population (203/1000 person years). Similarly, emergency department visits were also highest among the incident COPD population compared to prevalent COPD population and non-COPD population (866 incident versus 785 prevalent versus 328 non-COPD population) [4].

To curb the flow of previously undetected older individuals into acute care and emergency department utilization, it is imperative to identify these individuals to maximize available therapeutic options. Unfortunately, no clear guidelines exist for screening older individuals for COPD. Therefore, the purpose of the present study was to explore the utility of proactive identification of undetected or poorly controlled COPD in patients 75 years of age and older with a systematic office-based approach using standardized tools in a Canadian primary care setting. To guide further exploration of COPD in this population in the future, we also sought to compare the characteristics of patients with and without a documented diagnosis of COPD.

## 2. Methods

### 2.1. Patient Population

This study was a retrospective medical records review of patients who underwent screening through the Case Finding for Complex Chronic Conditions in Seniors 75+ (C5–75) Program at the Centre for Family Medicine Family Health Team (CFFM FHT) in Kitchener, Ontario, from April 1, 2013, to April 31, 2014. The CFFM FHT provides interprofessional primary care to 18 family practices with a combined patient base of 28,000 in urban and rural settings in Southwestern Ontario, Canada.

The goal of the C5–75 program is the proactive identification of persons at high risk for adverse health outcomes and high resource utilization to initiate preventive strategies aimed at reducing adverse health outcomes and improving health amongst these patients. Within this pilot project, all persons aged 75 years and older were systematically screened for frailty, HF, COPD, cognitive impairment, and falls risk; those who screened positive for these conditions were referred to appropriate health care personnel or teams for appropriate diagnosis and prophylactic or therapeutic management. Screening processes were developed based on existing screening recommendations and local expert opinion, as well as feasibility of implementation in the context of a busy community-based family practice setting. Screening was completed by trained nurses and results documented in patients' electronic medical record.

All patients within the CFFM FHT over 75 years of age, who were able to communicate in English, ambulatory, not acutely ill or distressed, willing to complete the screening, and attending a routine office visit were screened as part of the C5–75 program during the same visit as their scheduled appointment with their family physician. In some cases, screening was not completed due to patient preference, lack of time, or other logistical issues related to patient flow in the clinic.

### 2.2. COPD Case Finding

Case finding is a strategy for identifying individuals at risk for a specific condition, to provide screening and, if warranted, treatment. The Canadian Thoracic Society (CTS) guidelines and a recent systematic review on case-finding strategies [20] recommend COPD screening for smokers [2]; in this study, we targeted those with a 20-pack-year or greater smoking history, as have other studies [8, 19]. All patients with a smoking history of at least 20 pack-years were asked about respiratory symptoms based on CTS guidelines [2] for COPD screening (as shown below in COPD screening questions), regardless of documented history of COPD. A positive CTS screen is defined as a positive response to at least one of the five questions. For patients with a previous diagnosis of COPD, screening served as an opportunity to ensure optimal management and to identify additional suggestions for management, particularly in cases where spirometry had not been completed in several years. Patients with positive responses to the CTS screening questions were referred for postbronchodilator spirometry if this had not been performed within the preceding year. Spirometry was performed by a Certified Respiratory Educator (CRE) trained respiratory therapist consistent with guidelines for spirometry in primary care [21] and American Thoracic Society and European Respiratory Society [22] and interpreted by one of the authors (James Milligan). COPD was defined as the presence of a postbronchodilator ratio of forced expiratory volume in the first second of expiration to forced vital capacity (FEV1/FVC) of less than 0.7, indicating persistent airflow obstruction that is not fully reversible [1, 2]. For this study, the FEV1/FVC cut-off of 0.70 was chosen as it is currently referenced in major guidelines [1, 23] and used in other COPD screening studies [8, 19]. 

*COPD Screening Questions. *Canadian Thoracic Society Guideline [2] questions (each positive response receives a score of one, for a maximum score of 5) are as follows:

 Do you cough regularly?
 Do you cough up phlegm regularly?
 Do even simple chores make you short of breath?
 Do you wheeze when you exert yourself, or at night?
 Do you get frequent colds that persist longer than those of other people you know?

### 2.3. Procedures

The charts of all patients screened in the C5–75 program were reviewed, abstracting the following data: age, gender, previous diagnosis of COPD, HF, Coronary Artery Disease (CAD; myocardial infarction, angina, and coronary artery bypass graft), hypertension, diabetes, hyperlipidemia, atrial fibrillation, cognitive impairment, and osteoporosis, spirometry completed in the past year, smoking history of at least 20 pack-years, CTS screening results, referral and status of referrals for spirometry and results, and new diagnosis of COPD.

Approval for this study was obtained from the Hamilton Integrated Research Ethics Board of McMaster University.

### 2.4. Statistical Analysis

Descriptive statistics (means, frequencies) were generated for all variables. Pearson chi-square or Fisher's Exact test, as appropriate, was used to identify significant differences between patients previously diagnosed with COPD and those without a previous COPD on sample characteristics and screening results. *t*-test was used to identify group differences in age. Statistical significance was set at *p* < 0.05. Data were analyzed using SPSS software (IBM Corp., Version 23.0, 2015).

## 3. Results

### 3.1. Patient Characteristics

During the study period, 516 patients were screened in the C5–75 program. Characteristics of the population are presented in Table 1. Patients ranged in age from 74 to 96 years of age; over half were female. Over half had a history of hypertension; 11% had a previous diagnosis of COPD. Significantly more patients with a previous diagnosis of COPD had HF and CAD than those without a previous diagnosis of COPD.

### 3.2. COPD Case Finding

**Figure 1:**
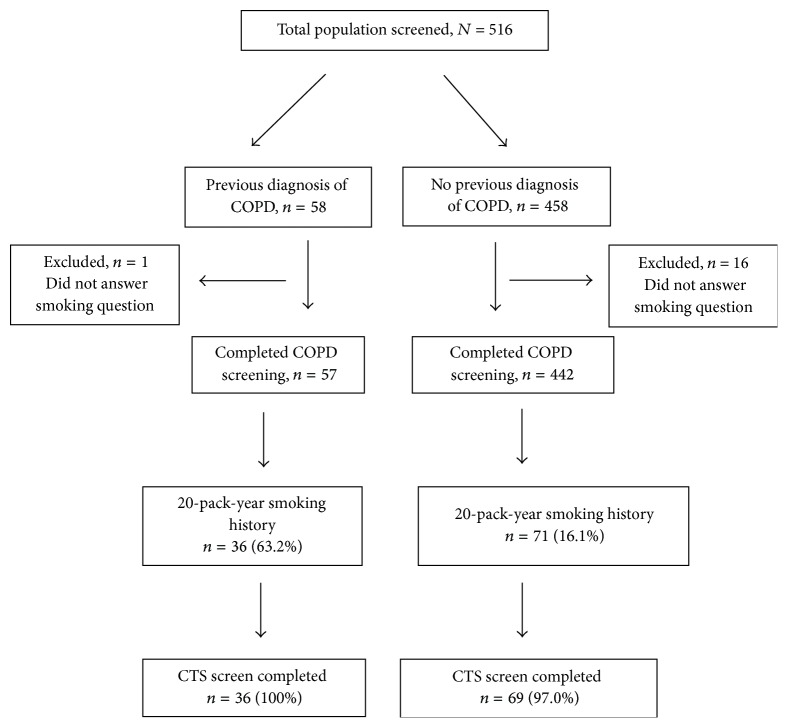
Response rates.

A total of 499 patients completed the screening questions; 17 were excluded as they did not have data on their smoking history (Figure 1).

A total of 107 patients (21%) were identified as having a 20-pack-year smoking history of whom 36 (34%) had a previous diagnosis of COPD (Table 2). Among patients with a 20-pack-year smoking history, significantly more patients who had a previous diagnosis of COPD also had a history of HF, hypertension, and spirometry in the previous year compared to those who were not previously diagnosed with COPD. Of the patients identified as having a 20-pack-year history of smoking, all but two patients were screened using the CTS questions for COPD.

### 3.3. Canadian Thoracic Society Screening Questions

Forty-four (42%) patients were positive on the CTS screen to one or more questions. Compared to those without a previous diagnosis of COPD, significantly more patients with a previous diagnosis of COPD screened positive to CTS screening questions; the most frequently positive responses being related to presence of regular coughing and shortness of breath, which were positive in over a third of the patients (Table 3).

### 3.4. Referrals for Spirometry and Impacts

Of the 44 patients that screened positive on the CTS, 30 patients were referred for spirometry, 14 of whom had a previous diagnosis of COPD; 14 patients were not referred for spirometry, 8 because they had completed spirometry within the previous year and in 6 cases it was not clear based on available documentation why they were not referred. In total, 21 spirometry assessments were completed. For nine of the patients who were referred for spirometry, the test was not completed because it had been completed within one year (*N* = 1), patients (*N* = 3) or physicians declined the assessment (*N* = 2), or the patient did not attend the appointment or died (*N* = 3). Of those who completed spirometry and who were not previously diagnosed with COPD (*N* = 11), four (36.4%) were newly diagnosed with COPD. Spirometry results were used to modify existing treatment (change in inhaled medication type or dose or change in inhalation techniques) in 71.4% (5/7) of patients previously diagnosed with COPD; these patients did not have spirometry assessments within the previous year.

## 4. Discussion

Case finding, screening, and detection of COPD among older adults are particularly significant given the high prevalence of this chronic condition among those over 65 years of age [5], higher health system utilization among older persons with COPD in comparison to younger persons [4], and given that it often goes undetected [6]. Relatively few studies have focused on case finding for COPD among older adults within primary care and there is currently limited understanding of how best to screen for COPD in older adults. This study explored the use of opportunistic case identification of older adults with COPD in a Canadian primary care setting, using systematic inquiry to identify symptoms suggesting individuals at highest risk who might benefit from diagnostic spirometry. Our study found that routine inquiry using the CTS recommended screening questions in persons aged 75 and older with at least 20-pack-year smoking history to be feasible in the context of a busy clinical setting and resulted in new spirometry-based diagnoses of COPD in 36% (4/11) of patients, and, in those with known diagnoses of COPD, positive screening and spirometry resulted in change to COPD treatment in 22% (2/9).

The barriers to effective diagnosis and management of COPD in general practice are complex. These include lack of specificity of symptoms such as dyspnea [24], presence of comorbid conditions [25], diagnostic confusion with asthma [9, 26], inadequate knowledge and training in COPD diagnosis and management [27, 28], and limited confidence in use and interpretation of spirometry results [29], resulting in significant under- and inaccurate diagnosis of COPD. In our study population, those with COPD had significantly more comorbidities of HF and CAD as compared to those without COPD; the overlap in symptoms for HF and COPD further complicates the diagnostic process [30]. These challenges, along with high rate of acute care utilization associated with COPD [4, 31, 32], highlight the need for an efficient, systematic approach to screening for appropriate management of this condition. Major guidelines support targeted case finding for COPD in primary care on the basis that early diagnosis of COPD combined with effective interventions can reduce the health burden associated with this disease [1, 2]. History and physical examination alone are limited in their ability to accurately identify persons with conditions such as COPD or HF [33–35]. Several studies have demonstrated the value of short screening questionnaires with spirometry [10, 17, 36] and have shown that primary care spirometry testing improves the accuracy of diagnosis and also results in significant improvements in management [37].

However, in our study population, one-third of the patients with a known diagnosis of COPD did not report a 20-pack-year or greater smoking history. This may be due to a number of factors; it may reflect inaccurate diagnosis, inaccurate self-report of smoking history, or that a cut-off of 20-pack-year smoking history is inappropriate as there is increasing recognition of the proportion of COPD cases among never-smokers, particularly females [38]. Restricting screening to smokers alone could potentially miss a significant number of cases. At least one-fourth of patients with COPD are nonsmokers [39]; one study suggests a 17% prevalence of COPD based on spirometry in subjects who never smoked or smoked less than 20 pack-years [9]. There may be individual variability in susceptibility to COPD [2]. Our results support not limiting COPD screening to those with a 20-pack-year smoking history and suggest the need for further exploration of the prevalence of COPD among nonsmoking older adults.

### 4.1. Limitations

Limitations to this study include our inability to identify the exact prevalence of COPD because not all patients positive on screening the CTS questions completed spirometry and because of the lack of long term follow-up of impact on care and the possibility of overdiagnosis of COPD based on fixed cut-off point for defining COPD. It could be suggested that as FEV1/FVC ratio falls with age [40], a lower limit of normal FEVI/FVC, in addition to the 0.70 cut-off, should be used to take into account age-related decline in this measure. There is currently some controversy regarding the most accurate cut-offs for spirometry to diagnose COPD; overdiagnosis and underdiagnosis can potentially occur using FEV1/FVC < 0.70 or lower limit of normal (LLN) alone and there is a suggestion that combining FEV1 < 0.08 will align with a more accurate diagnosis and outcome. In our sample population, restricting screening to those with a 20-pack-year or greater smoking history may have underestimated those at risk for COPD. Due to the retrospective nature of this study, quality control of the spirometry could not be assessed; however, as described, the spirometry was performed by two CRE respiratory therapists who have more training and expertise than might be encountered in some primary care performed spirometry. Of the 107 patients who were identified as having a 20-pack-year smoking history, 11 patients who were not previously diagnosed with COPD completed spirometry, four of whom were newly diagnosed with COPD. Although this may be considered a low yield, one must factor into the return on investment the well-documented high healthcare utilization costs associated with undiagnosed COPD [41] and that the development of proactive interventions to avert avoidable hospitalization begins with early accurate detection of the condition. However, future studies are currently underway to determine whether identification of frailty in primary care may improve the yield of screening processes to detect complex chronic conditions such as COPD in primary care. Further research is needed to assess the sensitivity and specificity of our screening process, impacts on health care utilization and cost-effectiveness, and the most efficient and feasible protocols for integrating spirometry in primary care.

## 5. Conclusions

As a systematic screen for COPD amongst elderly persons, use of CTS screening questions followed by spirometry for those identified at highest risk is feasible in the context of a busy primary care setting and identifies older adults with previously undiagnosed COPD. Further research should explore the value of not restricting screening to only those patients with a 20-pack-year smoking history and should be directed at developing and implementing an efficient primary care based program for the early detection and management of COPD in Canada as this may have major impact on the future burden of COPD.

## Figures and Tables

**Table 1:** Characteristics of all patients screened in the C5–75 program (*N* = 516).

Characteristics	Total sample (*N* = 516)	Previous diagnosis of COPD (*N* = 58)	No previous diagnosis of COPD (*N* = 458)	*p* value
Age, years, mean ± SD	80.8 ± 4.5	81.6 ± 4.7	80.7 ± 4.5	0.148
Gender, female, *n* (%)	291 (56.4)	23 (39.7)	268 (58.6)	0.006^*∗*^
Medical history prior to screening, *n* (%)				
Heart failure	34 (6.6)	10 (17.2)	24 (5.2)	0.002^*∗*^
CAD (MI, Angina, CABG)	119 (23.1)	21 (36.2)	98 (21.4)	0.010^*∗*^
Hypertension	264 (51.2)	26 (44.8)	238 (52.0)	0.306
Diabetes	118 (22.9)	14 (24.1)	104 (22.7)	0.807
Hyperlipidemia	188 (36.3)	22 (37.9)	166 (36.2)	0.801
Atrial fibrillation	54 (10.5)	10 (17.2)	44 (9.6)	0.074
MCI/dementia	61 (11.8)	6 (10.3)	55 (12.0)	0.707
Osteoporosis	133 (25.8)	20 (34.5)	113 (24.7)	0.108

COPD: chronic obstructive pulmonary disease; CAD: coronary artery disease; MI: myocardial infarction; CABG: coronary artery bypass graft; MCI: mild cognitive impairment.

^*∗*^Statistically significant (*p* < 0.05) differences between those previously diagnosed with COPD and those not previously diagnosed with COPD.

**Table 2:** Characteristics of patients with a 20-pack-year history of smoking (*N* = 107).

Characteristics	Total (*N* = 107)	Previous diagnosis of COPD (*N* = 36)	No previous diagnosis of COPD (*N* = 71)	*p* value
Age, years, mean ± SD	80.5 ± 4.7	81.5 ± 5.0	80.0 ± 4.4	0.108
Gender, female, *n* (%)	37 (34.9)	14 (38.9)	23 (32.4)	0.505
Medical history prior to screening, *n* (%)				
Heart failure	13 (12.1)	8 (22.2)	5 (7.0)	0.031^*∗*^
CAD (MI, Angina, CABG)	39 (36.4)	12 (33.3)	27 (38.0)	0.676
Hypertension	57 (53.3)	13 (36.1)	44 (62.0)	0.011^*∗*^
Diabetes	30 (28.0)	7 (19.4)	23 (32.4)	0.159
Hyperlipidemia	35 (33.6)	11 (30.6)	25 (35.2)	0.630
Atrial fibrillation	15 (14.0)	5 (13.9)	10 (14.1)	0.978
MCI/dementia	10 (9.3)	2 (5.6)	8 (11.3)	0.490
Osteoporosis	30 (28.3)	12 (33.3)	18 (25.4)	0.385
Spirometry in previous year	9 (8.4)	6 (16.7)	3 (4.2)	0.032^*∗*^

COPD: chronic obstructive pulmonary disease; CAD: coronary artery disease; MI: myocardial infarction; CABG: coronary artery bypass graft; MCI: mild cognitive impairment.

^*∗*^Statistically significant (*p* < 0.05) differences between those previously diagnosed with COPD and those not previously diagnosed with COPD.

**Table 3:** Results of the Canadian Thoracic Society screen for COPD screen for patients with a 20-pack-year history of smoking (*N* = 105).

Screening results	Total (*N* = 105) *n* (%)	Previous diagnosis of COPD (*N* = 36) *n* (%)	No previous diagnosis of COPD (*N* = 69) *n* (%)	*p* value
Negative CTS screen for COPD	61 (58.1)	13 (36.1)	48 (69.6)	0.001^*∗*^
Positive CTS screen for COPD^*∗∗*^	44 (41.9)	23 (63.9)	21 (30.4)	
CTS screening for COPD, positive screens for the following:				
Coughs regularly	23 (21.9)	13 (36.1)	10 (14.5)	0.011^*∗*^
Coughs up phlegm regularly	25 (23.8)	10 (27.8)	15 (21.7)	0.490
Being short of breath	31 (29.5)	20 (55.6)	11 (15.9)	0.001^*∗*^
Wheezes	12 (11.4)	9 (25.0)	3 (4.3)	0.003^*∗*^
Having frequent colds	2 (1.9)	1 (2.8)	1 (1.4)	0.636

COPD: chronic obstructive pulmonary disease; CTS: Canadian Thoracic Society screening questions [2].

^*∗*^Statistically significant (*p* < 0.05) differences between those previously diagnosed with COPD and those not previously diagnosed with COPD.

^*∗∗*^Positive response on at least one of the five CTS screening questions.
